# Adolescent Friendly Services: quality assessment with simulated users

**DOI:** 10.11606/s1518-8787.2020054001812

**Published:** 2020-03-30

**Authors:** Rosalila Pastrana-Sámano, Ileana Beatriz Heredia-Pi, Marisela Olvera-García, Midiam Ibáñez-Cuevas, Filipa De Castro, Aremis Villalobos Hernández, Maria del Pilar Torres-Pereda

**Affiliations:** I Centro de Investigación en Sistemas de Salud Instituto Nacional de Salud Pública de México CuernavacaMOR México Centro de Investigación en Sistemas de Salud . Instituto Nacional de Salud Pública de México . Cuernavaca , MOR , México; II Centro de Investigación en Salud Poblacional Instituto Nacional de Salud Pública de México CuernavacaMOR México Centro de Investigación en Salud Poblacional . Instituto Nacional de Salud Pública de México . Cuernavaca , MOR , México

**Keywords:** Qualitative research, sexual and reproductive health, Adolescent health, Patient Simulation

## Abstract

**OBJECTIVE:**

To assess the quality of adolescent friendly health services.

**METHODS:**

Qualitative assessment using the simulated user technique in first level clinics of Health Services of Morelos, Mexico, during 2018. Ten out of 17 facilities with non-exclusive adolescent friendly services were randomly selected. An additional facility with exclusive adolescent friendly services was included as an intensive subsample. Four adolescents served as simulated users interpreting different cases in the clinics. The total of 43 semi-structured exit interviews were conducted, and two nominal groups were made to assess the perceived quality from the adolescents’ perception of friendliness and experience. Thematic analysis of the data obtained was performed.

**RESULTS:**

Staff attitude was highlighted as a key element in the adolescents’ experience. Failures were found, such as the existence of bureaucratic barriers to access, lack of signage in clinics, lack of privacy and confidentiality, failure of physical examination during the appointment and lack of monitoring of the reasons for appointment. The exclusive clinic for adolescents offered more appropriate friendly services compared with nonexclusive clinics.

**CONCLUSION:**

Although the service is accessible in most of the clinics visited, it is still far from being friendly according to international recommendations. The exclusive clinic for adolescents stood out for having better structured mechanisms that can be implemented in nonexclusive clinics to improve the care process.

## INTRODUCTION

Globally, adolescents face problems such as pregnancy, early sexual activity, lack of knowledge and use of contraceptive methods, as well as an increased incidence of sexually transmitted infections (STI) that affect their well-being ^1^ . In Mexico, where 18.4% of the population is made up of adolescents ^2^ , it is essential to address the sexual and reproductive health (SRH) needs of this population group.

Prevention of teenage pregnancy is central, because during this stage the likelihood of dying from obstetric events increases ^3^ . Adolescent pregnancy is associated with school dropout, few job opportunities, early marriage, predisposition to poverty and can place young people in situations of insecurity and abuse. These conditions limit their personal, occupational and social development ^4^ . Although the problem of adolescent reproductive health affects both women and men in this age group, the consequences of adolescent pregnancy and the experience of the phenomenon itself are differentiated according to genre. Maternity has a disproportionate and very negative impact on adolescent women, which is directly related to gender inequalities and socio-cultural factors ^5^ .

In Mexico, although 98.2% of adolescents between the ages of 15 and 19 reported knowing some contraceptive methods, 69.2% of women who began sexual life before the age of 20 did not use any contraceptive method in their first intercourse ^6^ . The adolescent fertility rate in 2016 was 61 births per 1,000 adolescents from 15 to 19 years old, ranking first among countries of the Organization for Economic Cooperation and Development (OECD) ^7^ . In addition, the incidence rate of STI among adolescents increased between 2006 and 2012: Human Papilloma Virus (HPV) (from 5.01 to 7.97); HIV (from 0.76 to 1.63) and herpes (from 0.56 to 1.03) ^8^ .

Adolescents have been considered a healthy subset of the population. However, the increase in sexual and reproductive problems in this population has shown the need to provide adolescents with effective, appropriate and quality health services to assert their sexual and reproductive rights ^9 , 10^ . The World Health Organization (WHO) proposes the adolescent friendly health service (AFHS) model, which provides a space where adolescents feel safe and confident to come for advice and care, primarily in the area of sexual and reproductive health (SRH) ^11^ . This space should have empathetic staff trained in SRH and adolescent development issues ^1 , 9 , 12^ . Following these recommendations and given the importance of adolescent health, the AFHS model was adopted in Mexico in 2000. In 2017, there were 1,494 units with AFHS in the Ministry of Health (MH) nationwide, while in the state of Morelos there were 17 ^13^ . Despite the implementation of AFHS, the health indicators for this population group lead us to consider their effectiveness, since they do not meet the needs of the target population ^6 - 8^ . Programs that promote access and acceptance of SRH services for adolescents are more effective when the friendly approach is combined with their needs and expectations ^14^ . Strategies are needed to bridge the gap between the adolescent population and the health system, especially by making the male adolescent visible. Health programs are mainly focused on women in relation to reproductive issues ^15^ .

To assess the implementation of AFHS is relevant and necessary for measuring quality and establishing the impact on adolescent health ^8^ . Few studies assess these services ^16 - 19^ , as most are quantitative and were conducted from the perspective of providers. Therefore, a qualitative assessment, from the adolescents’ perspective, would allow us to understand their experience and identify barriers in the access, use and supply of services. However, adolescents are not always informed about the quality standards in AFHS.

There are various approaches to measure the quality of health services. The Figure shows the approaches outlined in the literature, as well as the dimensions used to measure the quality of the AFHS used in this study. This assessment proposes the use of simulated user methodology to assess the quality of AFHS by trained adolescents, making simulated visits, while health care providers are unable to change their behavior when they know they are being observed ^20^ . The objective of the study was to assess the quality of AFHS based on the dimensions of accessibility, opportunity, acceptability/adaptability, safety and continuity.

**Figure:**
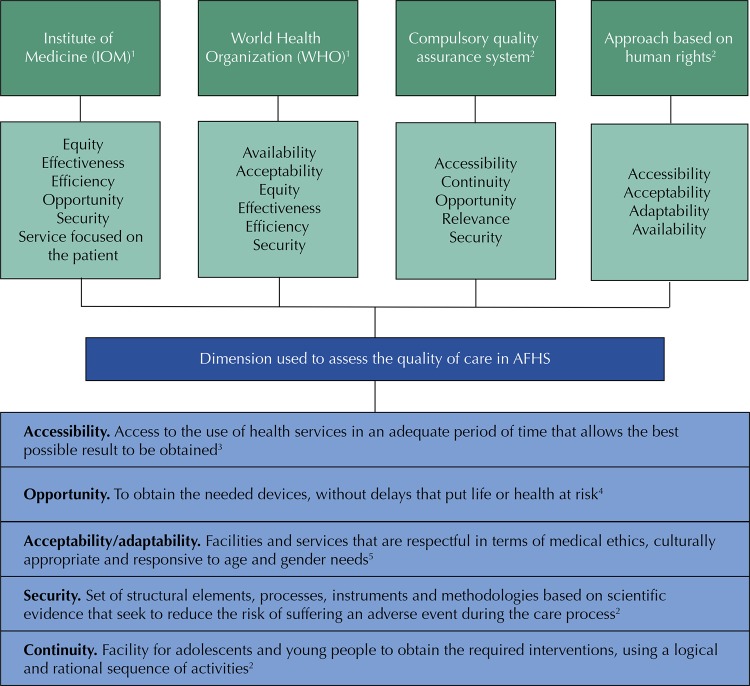
Dimensions to assess the quality of care of Friendly Services from different perspectives, Mexico 2018. 1. Saturno Hernández PJ. Métodos y herramientas para la realización de ciclos de mejora de la calidad de los servicios de salud. Cuernavaca, Morelos: Instituto Nacional de Salud Pública, 2015. 2. Fondo de Población de las Naciones Unidas. Servicios de salud amigables para adolescentes y jóvenes.: Un modelo para adecuar las respuestas de los servicios de salud a las necesidades de adolescentes y jóvenes de Colombia. Segunda. Bogotá: UNFPA, 2008. 3. Landini F, González-Cowes V, D’Amore E. Hacia un marco conceptual para repensar la accesibilidad cultural. Cadernos de saude publica 2014; 30(2):231–44. 4. Delgado-Bernal M, Márquez-Villarreal H, Santacruz-Varela J. La calidad de la atención a la salud en México a través de sus instituciones: 12 años de experiencia. La seguridad del paciente: eje toral de la calidad de la atención. Primera. México, D.F.: Secretaria de salud; 2012. 5. Organización Mundial de la Salud. Salud y derechos humanos. 2017. Available from: http://www.who.int/es/news-room/fact-sheets/detail/human-rights-and-health. AFHS: Adolescent Friendly Health Services

## METHODS

Qualitative assessment using simulated user methodology. The study scope was health centers located in Morelos, Mexico, that offered the AFHS model. Ten of the 17 MH clinics with AFHS in 2017 were randomly selected. The selection of nonexclusive centers for adolescents was adjusted by unit size, geographic location and volume of services in the last year. The Center for Comprehensive Adolescent Health Care (CAISA – *Centro de Atención Integral a la Salud del Adolescente* ), a clinic dedicated to serving the adolescent population exclusively and considered standard in AFHS, was included as an intensive type subsample ^21^ . A total sample of 11 clinics was obtained.

The technique of the simulated user is a form of participant and hidden observation that allows information to be obtained from the user experience and is an approach to the perceived satisfaction of the service ^20^ . It enable us to identify factors that make users decide to return to a health facility or not, due to the perceived quality of the service received ^20^ . This study assessed perceived quality in five dimensions of service friendliness: accessibility, opportunity, safety, acceptability/adaptability, and continuity ( Figure ).

Four profiles were created, one for each simulated user, which would be systematically interpreted during clinic visits. The profiles defined each adolescent’s history and the purpose of the appointment. Half of the profiles corresponded to females and half to males, and they were uniformly distributed between minors and adults (Supplementary material). To achieve theoretical data saturation ^22^ , each simulated user visited the 11 clinics in the sample, completing 44 visits in total. However, one user was denied care at one of the clinics.

Four adolescents aged 18 were selected and trained. They were chosen in late adolescence because they should have reached the legal age in order to obtain a paid job in the research project. The following criteria were considered for selection: ease of speech, good memory, ability to improvise and adapt to unexpected situations, that none of them had children, nor had used the AFHS before, seeking to create a homogeneous group and minimizing bias due to previous experience in the AFHS.

The training lasted 16 hours, addressing technical and ethical aspects of the study, additionally offering information about SRH in order to provide the four simulated users with homogeneous knowledge to assess the quality of the information offered in the clinics.

The semi-structured exit interview (SSI) was used to collect the data. The interview guide explored the dimensions of friendliness and contained specific case questions at the end. Interviews were conducted after each clinic visit and were carried out via telephone. They lasted approximately 30 minutes, were audiotaped and conducted by a member of the research team.

Two nominal groups ^23^ were formed with the simulated users as a mechanism for rigor and data quality, as well as to involve adolescents in the data analysis process. The groups were moderated, and audios were recorded by members of the research team.

The information collected via SSI and the nominal groups was transcribed *verbatim* (that is word for word). Thematic analysis was done ^24^ . We looked for themes, within the categories of analysis that allowed us to understand the phenomenon studied and that contained detailed and articulated explanations to the research questions.

The research protocol was approved by the ethics and research committee of the National Institute of Public Health, Mexico. Informed consent form was obtained from health authorities and health care providers. The study was totally anonymous, the names assigned were fictitious to protect confidentiality.

Psychological support was provided to adolescents to minimize the potential for emotional bias resulting from the development of their role and to minimize any unintended consequences of their participation.

## RESULTS

The total of 43 exit SSI were collected, the results found are presented in the five dimensions analyzed, the central findings with theoretical saturation and the exceptional or deviant ones are pointed out. Table 1 shows the testimonies that illustrate the results and those identified with codes that indicate the characteristics of origin and context of the testimony.

**Table 1:** Testimonies by dimension and type of finding, Mexico 2018.

Dimension	Testimonies	

	Theoretical Saturation	Exceptional
Accessibility	*Easy geographical access* **A1.** Claudia: Yes, yes, ***it was very easy for me, in fact, the bus passes right in front,*** I mean, it’s next door [bus stop] and you get off and there it is [the health center]. EP3 ^a^ _10 ^b^ _Woman ^c^ _ICM ^d^ *Free care* **A2.** Armando: ***They told*** *me* ***everything was totally free.*** EP1_10_Man_STI	*Unclear indications* **A3.** Claudia: […] ***I walked in and I didn’t know where I was,*** I just stood there for, like, five minutes ***until a nurse said, “What do you need?”*** […],”I want information about contraceptives”; ***and she said “ok, but you have to wait” but she didn’t say where, or anything.*** Second_ Nominal Group _Session01_P3_Woman *Conditioned care* **A4.** Laura: […] [ *The nurse* ] ***asked me if I had a policy, insurance, IMSS or ISSSTE*** [public insurers], ***and I said no. She told me to come at 7 in the morning and get a card, which would cost me 72 pesos*** so that I could see the doctor. […] EP2_07_Woman_ICU
Opportunity	*Prioritization of care* **O1.** *Claudia: […] a doctor asked me what I needed, and I told her that I wanted some information about contraceptives, and* ***she told me to wait a little while, I waited less than a minute and she talked to me right away*** *[…] EP3_03_Woman_ICM* **O2.** Laura: [The doctor] ***told me to wait a little while*** because there were many patients and not much staff, but she ***was going to do something to find somebody talk to me*** [...] ***and I waited.*** EP2_11_Woman_ICU *Failure in the interrogation and detection of risk factors* **O3.** Julio: ***For example, in the case I was involved in*** (doubt about mechanisms of HIV transmission) [...] ***they should have said, asked me more intimate things, but only in some places they asked me.*** […]. […].For example, ***in the majority, they asked me if I had risky practices, I told them that I did not, and they did not ask me anything else. They never specified what a risky practice was.*** Second Nominal Group_Session01_P4_Man	*Long waiting time* **O4.** Laura: ***Well, they made me wait, I had turn 12, I was the last one, I waited, I’m not lying, not lying for you, for three hours*** […].For me it was too upsetting […] EP2_01_Woman_ICU *Delays in care* **O5.** Armando: She told me [at the reception] that [friendly services] ***were not available,*** because the doctor was not there, ***and I should come back on Tuesdays and Thursdays for that kind of thing, specific for young people.*** EP1_08_Man_STI
Acceptability/ Adaptability	*Care in inappropriate space* **B1.** Laura: At the desk [located in the waiting room] ***she attended me and there were patients waiting for their appointment*** [...], and I was too embarrassed to speak, ***I was too embarrassed to play my role,*** […] I felt very uncomfortable ***with other people listening to me.*** EP2_07_Woman_ICU *Use of teaching materials in the appointment* **B2.** Julio: Well, ***it was a bit of a large office, it had many posters*** and articles for dynamics, about contraceptives, reproductive devices, in other words, ***it was appropriate to give an explanation of anything.*** EP4_09_Man_IHIV	*Safeguarding of confidentiality* **B3.** Laura: “ ***Don’t think that I’m running to your parents*** and telling them that you’re having sex or anything like that, ***You come here, and everything is confidential, so don’t feel embarrassed*** ” […] EP2_11_Woman_ICU
Security	*Complete and clear information* **S1.** Claudia: She ***only took out the condom,*** in fact she didn’t have a female one, but explained to me with the male condom, and ***it was with her hands*** she didn’t have something like a dummy […], ***but she explained it to me.*** EP3_09_Woman_ICM *Physical Examination Failures* **S2.** Armando: And then ***it wasn’t a very nice care because she didn’t weigh, didn’t measure me, nothing.*** EP1_01_Man_STI *Negotiating condom use* **S3.** Armando: ***She told me to talk to the girl I’m dating,*** [...] ***and tell her*** “I know you like it when we do it unprotected... ***we have to use that [condom]*** to protect ourselves, to avoid an unwanted pregnancy […]” EP1_06_Man_STI	*Failure of the information provided* **S4.** Laura: […] ***I have been instructed many times about how to use a condom and she lost some details when she showed me.*** And I noticed because I already knew. […]. EP2_07_Woman_ICU **S5.** Claudia: *She didn’t help me as much,* ***she explained the contraceptives a little but not completely as others did,*** *I think* ***if I hadn’t already gone to other health centers*** *[…]* ***I think I wouldn’t have understood anything she said.*** EP3_07_Woman_ICM
Continuity	*Possibility to return when necessary* **C1.** Julio: Well, ***she told me that when I wanted, I could come back, that’s why it is a friendly service, and there was no problem, the doors were open for me.*** EP4_03_Man_IHIV	*Means of communication between provider and patient* **C2.** Julio […] ***I was surprised that she invited me... that she could give me any appointment of continuity in the places where she works,*** *[... the psychologist]* ***told me I can go with her to the place where she works, she gave me her telephone number.*** EP4_08_Man_IHIV *Offer of various services* **C3.** Laura: *But* ***they told me that, if I wanted to, I could make an appointment at the reception desk for the dentist and the nutritionist.*** EP2_06_Woman_ICU

^a^ EP1= Profile 1, EP2= Profile 2, EP3= Profile 3, EP4= Profile 4

^b^ 01 a 11= Health establishment code

^c^ Sex of participant

^d^ STI= sexually transmitted infections, ICU= information about condom use, ICM= information about contraceptive methods and IHIV= information about HIV.

### Accessibility

In general, easy access was reported due to the geographical location and wide availability of public transport with access to the units. Clinics with adequate internal signage made it easy to locate the service. This was perceived as pleasant since they did not have to ask the staff about the service, while safeguarding the confidentiality of the visit. In most facilities, free care was provided only for the AFHS (Testimonies A1 and A2, Table 1 ).

When units are inadequate or do not have signposting, they have to ask for service information. But instructions were sometimes unclear, resulting in appointment delays. In a minority of cases, there were administrative barriers to receiving care: appointments were conditioned on affiliation to health insurance and fees were charged per appointment or contraceptive method (Testimonies A3 and A4).

### Opportunity

The waiting time was less than 30 minutes in most units. Despite this, a clinic was found where they waited approximately four hours for care. One participant pointed out that she only waited because she was playing the role of a simulated user, making it clear that she would have left otherwise (Testimonies O1, O2 and O4).

Most units did not have a mechanism for appointments or rescheduling, leaving open the possibility of treatment when they require the service, but not guaranteeing assistance when they return (Testimony O5).

The staff tried to find a way to provide care or information that would solve the adolescent’s problem. Inadequate practices in detecting risky sexual behavior were recorded. Simulated users found that questioning was incomplete and observed that some providers were not comfortable interrogating adolescents about their sexual behavior (Testimony O3).

Although a minority, there were clinics where care was not provided for reasons such as: unavailability of AFHS, service saturation, lack of professionals, inadequate hours, or it was simply alleged that care could not be provided, suggesting patients to return another day (Testimony O5).

The exclusive center for adolescents had extended opening hours (morning and afternoon). In addition, they had mechanisms for scheduling and controlling appointments, different from the nonexclusive centers, where the service was offered only in the morning, with no appointment control.

### Acceptability/adaptability

All the services had clinics that provided care to adolescents (Testimony B2). Most of the simulated users were treated in a clinic, but they were not always exclusive to AFHS, or provided a privacy environment (Testimony B1).

The attitude of the staff was considered central to the care process. A good attitude was defined as friendly, respectful, trustworthy, smiling, listening without interruption and showing interest. These elements generated trust and confidence in the adolescents, who stated they would recommend the service. A bad attitude was defined as unfriendly, rude, uncouth gestures, uncomfortable looks or judgments about the users’ sexual practices, making they feel reprehended, misunderstood and therefore not willing to return.

The confidentiality policy was presented to teenagers in an exceptional way (Testimony B3). However, the adolescents expressed the belief that when staff showed a good attitude, their information would be protected, or threatened when staff showed a bad attitude. Both privacy and confidentiality were violated by waiting room care, simultaneous care of patients in the same office, interruptions during appointment, and leaving the door open during care (Testimony B1).

At the exclusive center, the confidentiality policy was regularly mentioned, it was explained that information would only be revealed in cases in which their lives were at risk. Their sexual rights were also discussed; in nonexclusive services it did not happen.

### Security

The training of the users allowed them to identify errors or omissions in the information provided, which was not always truthful, useful or timely (Testimonials S4 and S5). There was variability according to the topic consulted, with contraceptive method and condom use being best explained, while STI/HIV counseling was the worst (Testimony S1). The use of didactic material such as models, audiovisual material and plastic models was perceived as positive, since it facilitated learning.

The offer of diagnostic studies depended on the case of appointment, mainly on the case of suspected HIV, in which rapid tests were more frequently offered. The information provided about diagnostic studies was unclear and confusing. Physical examination was an unusual practice (Testimony S2). In cases when it was done, it consisted of weight, height and blood pressure measurements. On rare occasions, ears and eyes were checked. The physical examination was more complete in the center exclusive for adolescents.

Health care providers, regardless of the reason for appointment, emphasized condom use with the partner as the only method that prevents unwanted pregnancy and STI/HIV (Testimony S3).

### Continuity

There was no continuity in the reason for appointment via follow-up appointments, although the opportunity was left open to return when they needed care (Testimony C1). Clinics did not offer services in addition to SRH, and few of them invited users to attend SRH talks or workshops. At the exclusive center, monitoring was provided and services such as nutrition and dental care were offered.

There were two exceptional cases in which providers, concerned about the health of users, provided a personal telephone number or information about their working hours to follow the case. This element was highly appreciated by the adolescents (Testimony C2).

The continuity dimension presented one of the important differences between nonexclusive and exclusive clinics for adolescents. In the exclusive clinics, the supply of other services in the health unit was consistently observed (Testimony C3). Table 2 summarizes the main findings, as well as the differences between exclusive and non-exclusive services found by the simulated users.

**Table 2:** Summary of key findings and differences between clinics with exclusive and nonexclusive services, Mexico 2018

Dimension	Accessibility	Opportunity	Acceptability/ Adaptability	Security	Continuity
Central findings	Easy geographical access Inadequate signage Free service, in some cases conditioned	Acceptable waiting times Inefficient detection practices Lack or deficiency of appointment control mechanisms	Variability of the place of care Lack of privacy protection Confidentiality is not always respected	Variability of information according to subject and personnel Lack of physical examination Limited supply of diagnostic studies Condom negotiation recommendation	Lack of monitoring Limited supply of complementary services
Differences between exclusive and nonexclusive services	No	Extensive service schedule (morning and afternoon) in specialized center, nonexclusive centers do not comply schedules offered. Agenda and control of appointments in exclusive center	In exclusive services they talk about the confidentiality policy and about sexual rights differently from nonexclusive services	More complete physical exploration in the exclusive service	Follow-up to the reason for appointment and offering of complementary services in the exclusive center, unlike the nonexclusive centers Partner or friends are invited to return as companions to the exclusive service

## DISCUSSION

There are areas for improvement to ensure that the quality of care in AFHS is optimal and effective, particularly in nonexclusive AFHS. Based on the experience of simulated users, services are geographically accessible. However, our study does not present disaggregations by type of locality, so it differs from other studies that point to difficult access in rural areas, as noted by Regmi et al. ^25^ . Most units do not have signs with schedules, days, cost of service, or signage of the service, resulting in users receiving indications from staff, in line with other studies that report lack of signage and unclear indications from staff ^16 , 17^ .

In Mexico, the friendly service is free. However, there were cases in which procedures such as charging and health insurance requirements were a barrier. Charging for the service is a significant and documented barrier to accessing health services ^16^ . If adolescents incur costs for using services, this reduces the chances of them returning to use the service or applying for contraception. In addition, adolescents avoid services using family health insurance for fear that their parents will find out ^5^ .

Waiting time was not a barrier for most clinics. However, there were exceptions that caused adolescents to want to leave and not return to a clinic. Schriver points out that waiting time needs to be optimized because it is one of the main constraints on attending services ^26^ , a barrier that may be even more critical for male adolescents, for whom the need for quick and direct service is important ^27^ . There were few cases when care was denied, as found by De Castro et al. and Sykes, who point out that professionals denied care to adolescents and invited them to return another day ^17 , 28^ . The clinics do not have clear mechanisms for scheduling visits, which is a central problem for continuity. Inviting adolescents to return does not guarantee that they will be assisted when they return, further wasting the opportunity to provide information and meet the health needs of adolescents.

Lack of privacy is the main barrier users faced when they use the services. The units have spaces designed to provide care, but the infrastructure is not always optimal to guarantee privacy ^16 , 17 , 29 - 31^ . Users prefer units with an exclusive space for adolescents, but most clinics do not have it. Villalobos et al. specify that 78.3% of the clinics do not have exclusive spaces, and this element is registered in the literature as central ^16 , 17 , 29 - 32^ , especially for males, who fear their masculinity will be impaired by being exposed to their community, because of SRH care ^5^ .

Privacy and confidentiality are key to avoid that users feel embarrassed or unmotivated to express their doubts ^28^ . Therefore, they constitute crucial elements in the satisfaction perceived by adolescents, since an atmosphere of distrust is generated when these characteristics are not present.

Physical examination is not a common practice, an element that diminishes the quality of AFHS, which must provide comprehensive care and systematize clinical examination during counseling ^9^ .

Users point the need to provide clear, truthful and specific information, as found in a previous study ^28^ . The importance given by staff to condom use as a method of preventing STI and unplanned pregnancies and the use of educational materials were important for counseling. This favored a dynamic appointment and facilitated the understanding of adolescents as pointed out by De Castro ^28^ .

There was no monitoring after appointments in most units, even when diagnostic tests for STI/HIV were required, although the user was invited to return when needed. With the exception of the exclusive center, the rest of the facilities do not promote complementary services among users.

A key finding for the quality perceived by adolescents is the staff’s attitude. Most had a respectful attitude, but we found cases when staff were critical, made moral judgments or showed an unprofessional behavior. Negative staff attitudes have been identified as one of the main barriers to service quality ^16 , 17 , 30 , 31^ .

Although the exclusive services were perceived as successful, by providing friendly care, infrastructure and resource requirements limit their replication in a general way. Friendly services in public clinics to people without social security are the predominant model in the state. The findings of this study point out areas of opportunity for these adolescents and document good care practices, according to the resources available in each clinic.

In this study, the simulated user methodology enabled us to obtain objective information from a trained and standardized fictitious client ^20 , 33^ . The experiences of the simulated users during the search and obtaining of health services generated evaluative data, the result of a comparison and contrast exercise that can be differentiated from the poor knowledge about quality standards of real users. This generates valuable evidence for the discussion of quality studies from the perspective of the users ^34^ .

Our findings and the analyses presented are observational and do not seek to identify causality among the phenomena studied. The methodology carried out a critical assessment and facilitated observation without altering the behavior of the service provider when being examined ^35^ .

No differentiation was made between urban and rural areas, due to the geographical location of the centers, and all possible cases of appointment were not addressed. Although there were two male and two female simulated users, no specific attempt was made to understand differences related to sex or sexual preference of users, but to the dimensions of the quality of the service. This study contributed by identifying findings that may go unnoticed by a common user, due to lack of knowledge about the topic consulted.

## CONCLUSION

Although most of the establishments visited by the simulated users refer to having a friendly service, they are still far from meeting the characteristics of friendliness according to international recommendations. This study reports important findings about quality during the care process. Services do not always provide integral care to adolescents, forgetting prevention and continuity of care once the immediate reason for appointment is addressed. In addition, although there were few cases, care is still conditioned on payment for services. Due to the evidence in the literature about disparities in the experiences and consequences of adolescent pregnancy, it is necessary for services to incorporate a gender focus that allows the active use of sexual and reproductive health services by males.

Differences were found between adolescent-only and non-exclusive clinic-based facilities. The perception of the simulated users showed that the care in the exclusive centers is better and more complete, offering various services, regardless of the reason for appointment. Although it is not possible for the health system to have exclusive friendly services in all cases, adaptations are necessary to allow current services to adopt strategies that make it possible for nonexclusive services to provide a service comparable to that of the exclusive center. It was best evaluated by the adolescents when indicated that they could return if they required care. It is a fact that the number of clinics providing adolescent health services has increased, but improvements are needed to achieve quality care.

Strategies are needed to improve good practices and quality of health care for adolescents, including the development of activities in communities, involving schools and parents, to attract and engage adolescents before they become sexually active. In addition, awareness raising and training for administrators and health personnel in the care of adolescents are essential, so that prejudices and social norms do not permeate the care offered to this population.
