# Supplementary material for: Adolescent Friendly Services: quality assessment with simulated users
Source: Rev Saude Publica. 2020 Mar 30;54:36. doi: 10.11606/s1518-8787.2020054001812 (PMC7112745; doi:10.11606/s1518-8787.2020054001812)
Supplement: Translation

# Translation: Servicios Amigables para Adolescentes: evaluación de la calidad con usuarios simulados

**Authors:** Rosalila Pastrana-Sámano, Ileana Beatriz Heredia-Pi, Marisela Olvera-García, Midiam Ibáñez-Cuevas, Filipa De Castro, Aremis Villalobos Hernández, Maria del Pilar Torres-Pereda

**Affiliations:** I México Centro de Investigación en Sistemas de Salud. Instituto Nacional de Salud Pública de México. Cuernavaca, MOR, México; II México Centro de Investigación en Salud Poblacional. Instituto Nacional de Salud Pública de México. Cuernavaca, MOR, México

**Keywords:** Investigación cualitativa, salud sexual y reproductiva, Salud de los adolescentes, Paciente simulado

## Abstract

**OBJETIVO:**

Evaluar la calidad de los servicios de salud amigables para adolescentes.

**MÉTODOS:**

Evaluación cualitativa utilizando la técnica de usuario simulado en clínicas de primer nivel de los Servicios de Salud de Morelos, México durante 2018. Se seleccionaron aleatoriamente 10 de 17 establecimientos con servicios amigables no exclusivos para adolescentes. Se incluyó adicionalmente un centro con servicios amigables exclusivo para adolescentes como submuestra de tipo intensivo. Cuatro adolescentes fungieron como usuarios simulados interpretando diferentes casos de consulta en las clínicas. Se realizaron 43 entrevistas semiestructuradas de salida y se hicieron dos grupos nominales para evaluar la calidad percibida a partir de la percepción de amigabilidad y la experiencia de los adolescentes. Se realizó análisis temático de los datos obtenidos.

**RESULTADOS:**

La actitud del personal destacó como un elemento clave para la experiencia de los adolescentes. Se encontraron fallas como la existencia de barreras burocráticas para el acceso, falta de señalamientos en las clínicas, falta de privacidad y confidencialidad, fallas en la exploración física durante la consulta y falta de seguimiento de los motivos de consulta. La clínica exclusiva para adolescentes ofreció servicios amigables más adecuados en comparación con las clínicas no exclusivas.

**CONCLUSIÓN:**

Aunque en la mayoría de los establecimientos visitados el servicio es accesible, aun distan de cumplir con las características de amigabilidad de acuerdo con las recomendaciones internacionales. La clínica exclusiva para adolescentes destacó al contar con mecanismos mejor estructurados que pueden ser implementados en clínicas no exclusivas para mejorar el proceso de atención.

## INTRODUCCIÓN

Globalmente, los/as adolescentes enfrentan problemas como embarazo, inicio de vida sexual a edad temprana, desconocimiento y falta de uso de métodos anticonceptivos, así como incremento de la incidencia de infecciones de transmisión sexual (ITS) que afectan su bienestar ^1^ . En México, donde el 18.4% de la población total corresponde a adolescentes ^2^ , resulta primordial atender las necesidades en salud sexual y reproductiva (SSR) de este grupo poblacional.

La prevención del embarazo adolescente es central, debido a que se incrementa la probabilidad de morir por eventos obstétricos en esta etapa ^3^ .El embarazo en la adolescencia está relacionado con deserción escolar, pocas oportunidades laborales, uniones tempranas, predisposición a la pobreza y puede colocar a los jóvenes en situaciones de inseguridad y maltrato. Eso limita su desarrollo personal, laboral y social ^4^ . Si bien la problemática de la salud reproductiva de los adolescentes afecta tanto a mujeres como a hombres de este grupo de edad, las consecuencias del embarazo en la adolescencia y la experiencia misma del fenómeno son diferenciadas de acuerdo al género. La maternidad tiene un impacto desproporcionado, y muy negativo, en las adolescentes mujeres, que está directamente relacionado con desigualdades de género y factores socioculturales ^5^ .

En México, aun cuando el 98.2% de los adolescentes entre 15 y 19 años declaran conocer algún método anticonceptivo, 69.2% de las mujeres que iniciaron vida sexual antes de los 20 años no utilizó ningún método anticonceptivo en su primera relación sexual^6^. La tasa de fecundidad en adolescentes para 2016 fue de 61 nacimientos por cada 1,000 adolescentes de 15 a 19 años, ocupando el primer lugar entre países de la Organización para la Cooperación y el Desarrollo Económico (OCDE)^7^. Adicionalmente, se incrementó la tasa de incidencia de ITS en adolescentes entre el 2006 y 2012, principalmente: Virus de Papiloma Humano (VPH) (de 5.01 a 7.97); VIH (de 0.76 a 1.63) y herpes (de 0.56 a 1.03)^8^.

Los adolescentes han sido considerados como un subconjunto saludable de la población. Sin embargo, el incremento de problemas sexuales y reproductivos en esta población ha mostrado la necesidad de otorgar a los adolescentes servicios de salud efectivos, apropiados y de calidad para hacer valer sus derechos sexuales y reproductivos^9 , 10^. La Organización Mundial de la Salud (OMS) propone el modelo de servicio de salud amigable para adolescentes (SSAA), que brinda un espacio en donde los adolescentes se sienten seguros y en un ambiente de confianza para acudir a recibir asesoría y atención, primordialmente en materia de salud sexual y reproductiva (SSR)^11^. Ese espacio debe tener personal empático y capacitado en temas de SSR y desarrollo adolescente^1, 9 , 12^. Siguiendo estas recomendaciones y dada la importancia que tiene la salud de los adolescentes, se adoptó el modelo SSAA en México desde el año 2000. En 2017, existían 1,494 unidades con SSAA en la Secretaría de Salud (SSA) a nivel nacional, mientras en el estado de Morelos existían 17^13^. Pese a la implementación de SSAA, los indicadores en salud para este grupo poblacional nos conducen a reflexionar sobre la efectividad de los mismos, al no lograr resolver las necesidades de la población objetivo^6^. Los programas que promueven el acceso y la aceptación de los servicios de SSR para adolescentes son más efectivos cuando el enfoque amigable se combina con las necesidades y expectativas de la demanda^14^. Se requieren desarrollar estrategias que acorten las distancias entre la población adolescente y el sistema de salud, particularmente haciendo visible al adolescente masculino. Los programas de salud están enfocados principalmente hacia las mujeres en relación a temáticas reproductivas^15^.

Evaluar la implementación de los SSAA es relevante y necesario para medir la calidad y establecer el impacto en la salud de los adolescentes^8^. Son pocos los estudios que evalúan estos servicios^16^, y la mayoría es de tipo cuantitativo y se realiza desde la perspectiva de los prestadores. Por lo tanto, realizar una evaluación cualitativa, desde la perspectiva adolescente, permitiría comprender la experiencia e identificar barreras en el acceso, utilización y oferta de servicios. Sin embargo, los adolescentes no siempre conocen los estándares de calidad en los SSAA.

Existen diversas aproximaciones para medir la calidad de los servicios de salud. La Figura muestra enfoques planteados en la literatura, así como las dimensiones utilizadas para medir la calidad de los SSAA utilizadas en este estudio. Esta evaluación plantea la utilización de metodología del usuario simulado para evaluar la calidad SSAA a través de adolescentes capacitados, realizando visitas simuladas, sin que los prestadores de salud puedan modificar su comportamiento al saber que están siendo observados^20^. El objetivo del estudio fue evaluar la calidad de los SSAA con base en las dimensiones de accesibilidad, oportunidad, aceptabilidad/adaptabilidad, seguridad y continuidad.

**Figura:** Dimensiones para evaluar la calidad de la atención de los Servicios Amigables desde diferentes perspectivas, México 2018. 1 Saturno Hernández PJ. Métodos y herramientas para la realización de ciclos de mejora de la calidad de los servicios de salud. Cuernavaca, Morelos: Instituto Nacional de Salud Pública, 2015. 2 Fondo de Población de las Naciones Unidas. Servicios de salud amigables para adolescentes y jóvenes.: Un modelo para adecuar las respuestas de los servicios de salud a las necesidades de adolescentes y jóvenes de Colombia. Segunda. Bogotá: UNFPA, 2008. 3 Landini F, González-Cowes V, D’Amore E. Hacia un marco conceptual para repensar la accesibilidad cultural. Cuadernos de salud pública 2014; 30(2):231–44. 4 Delgado-Bernal M, Márquez-Villarreal H, Santacruz-Varela J. La calidad de la atención a la salud en México a través de sus instituciones: 12 años de experiencia. La seguridad del paciente: eje toral de la calidad de la atención. Primera. México, D.F.: Secretaria de salud; 2012. 5 Organización Mundial de la Salud. Salud y derechos humanos. 2017. Disponible en: http://www.who.int/es/news-room/fact-sheets/detail/human-rights-and-health. SSAA: Servicios de Salud Amigables para Adolescentes

## MÉTODOS

Evaluación cualitativa utilizando metodología de usuario simulado. El universo de estudio fueron establecimientos de salud en el estado de Morelos, México, que ofertaran el modelo de SSAA. Del universo de 17 clínicas de la SSA con SSAA en el 2017, se eligieron 10 de forma aleatoria. La selección de centros no exclusivos para adolescentes se ajustó por el tamaño de la unidad, ubicación geográfica y volumen de servicios en el último año. Se incluyó como submuestra de tipo intensivo^21^al Centro de Atención Integral a la Salud del Adolescente (CAISA), clínica dedicada a atender población adolescente de forma exclusiva y considerada como estándar de oro en los SSAA. Se obtuvo una muestra total de 11 clínicas.

La técnica del usuario simulado es una forma de observación participante y oculta que permite obtener información desde la experiencia de los usuarios y constituye un acercamiento a la satisfacción percibida del servicio^20^. Permite identificar factores que hacen que los usuarios decidan regresar o no a un establecimiento de salud, debido a la percepción de la calidad del servicio recibido^21^. Mediante ella se evaluó la calidad percibida en cinco dimensiones de amigabilidad de los servicios: accesibilidad, oportunidad, seguridad, aceptabilidad/adaptabilidad y continuidad ( Figura ).

Se crearon cuatro perfiles, uno para cada usuario simulado, que serían interpretados sistemáticamente durante las visitas a las clínicas. Los perfiles definieron la historia de cada adolescente y el objetivo de la consulta. La mitad de los perfiles correspondían a mujeres y la mitad a varones, repartiéndose de forma homogénea entre menores y mayores de edad (Material suplementario). Para lograr la saturación teórica de datos^22^, cada usuario simulado visitó las 11 clínicas de la muestra, realizando un total de 44 visitas. Sin embargo, se negó la atención a una usuaria en una de las clínicas.

Se seleccionaron y capacitaron cuatro adolescentes de 18 años. Se escogieron en la etapa tardía de la adolescencia debido a que, al obtener un trabajo remunerado en el proyecto de investigación, debían contar con la mayoría de edad. Se consideraron los siguientes criterios para el reclutamiento: facilidad de palabra, buena memoria, capacidad de improvisar y adaptarse a situaciones no previstas, que ninguno tuviera hijos, ni hubiera utilizado antes los SSAA, buscando crear un grupo homogéneo y minimizando sesgos debido a la previa experiencia en los SSAA.

La capacitación duró 16 horas, abordando aspectos técnicos y éticos del estudio, ofreciendo adicionalmente información sobre SSR para que los cuatro usuarios simulados contaran con conocimientos homogéneos para poder evaluar la calidad de la información ofertada en las clínicas.

Se utilizó la entrevista semiestructurada (ESE) de salida para recolectar los datos. La guía de entrevista exploró las dimensiones de amigabilidad y contenía preguntas específicas del caso al final. Las entrevistas se llevaron a cabo después de cada visita a las clínicas y fueron realizadas vía telefónica. Duraron aproximadamente 30 minutos, fueron audio-grabadas y dirigidas por un miembro del equipo de investigación.

Se realizaron dos grupos nominales^23^con los usuarios simulados como mecanismos de rigor y calidad de datos, así como para lograr la participación de los adolescentes en el proceso de análisis de datos. Los grupos fueron facilitados y audio-grabados por miembros del equipo de investigación.

La información recabada mediante las ESE y los grupos nominales fue transcrita *verbatim* (es decir palabra por palabra). Se llevó a cabo análisis temático^24^. Al interior de las categorías de análisis, se buscaron temas que permitieron comprender el fenómeno estudiado y contenían explicaciones detalladas y articuladas frente a las preguntas de investigación.

El protocolo de investigación fue aprobado por el comité de ética e investigación del Instituto Nacional de Salud Pública, México. Se obtuvo el consentimiento informado de autoridades de salud y prestadores de los servicios de salud. El estudio fue totalmente anónimo, los nombres asignados eran ficticios para resguardar la confidencialidad.

Se brindó apoyo psicológico a los adolescentes para minimizar el potencial sesgo derivado de aspectos emocionales, consecuencia del desarrollo de su rol, y minimizar cualquier consecuencia no intencionada de su participación.

## RESULTADOS

Se recolectaron 43 ESE de salida, los resultados encontrados se presentan en las cinco dimensiones analizadas, se señalan los hallazgos centrales con saturación teórica y los excepcionales o desviantes. Los testimonios que ilustran los resultados se presentan en la Tabla 1 y son identificados con códigos que señalan las características de origen y contexto del testimonio reportado.

**Tabla 1:** Testimonios por dimensión y tipo de hallazgo, México 2018.

Dimensión	Testimonios	

	Saturación teórica	Excepcionales
Accesibilidad	*Fácil acceso geográfico* **A1.** Claudia: Sí, sí, **me fue muy fácil, de hecho, la ruta pasa exactamente enfrente** , o sea está al ladito y te bajas y ahí está luego, luego [el centro de salud]. EP3^a^_10^b^_Mujer^c^_IMA^d^ *Atención gratuita* **A2.** Armando: Me **dijeron que todo era totalmente gratuito** . EP1_10_Hombre_ITS	*Indicaciones poco claras* **A3** . Claudia: […] **Entré y no sabía dónde** , me quedé parada como por cinco minutos **hasta que una enfermera me dijo “¿Qué necesitas?** ” [ ], “quiero información acerca de anticonceptivos”; **y me dijo “ok, pero tienes que esperar” pero no me dijo dónde, ni nada** . Segundo_Grupo_Nominal_Sesion01_P3_Mujer *Atención condicionada* **A4.** Laura: […] **Me preguntó** *[la enfermera]* **que, si tenía póliza, seguro, IMSS o ISSSTE; y le dije que no; me dijo que llegará a las 7 de la mañana y que sacará una ficha, que me iba a costar 72 pesos** para que me diera consulta el doctor […] EP2_07_Mujer_IUC
Oportunidad	*Priorización en la atención* **O1.** *Claudia: [ ] una doctora me preguntó qué era lo que necesitaba, y le dije que quería un poco de información sobre anticonceptivos, y* **me dijo que me esperara tantito, me esperé como menos de un minuto y ya me habló** *[…] EP3_03_Mujer_IMA* **O2.** Laura: **Que me esperara tantito** porque había muchos pacientes y no había mucho personal; pero **que ahorita iba a hacer lo posible para que me atendieran** […] **y me esperé** . EP2_11_Mujer_IUC *Fallas en el interrogatorio y detección de factores de riesgo* **O3.** Julio: **Por ejemplo, en el caso que yo llevaba** (duda sobre mecanismos de transmisión de VIH) [ ] **tenían que haber dicho, preguntarme cosas más íntimas, pero solamente en algunos lugares me lo preguntaron** . […]. Por ejemplo, **en la mayoría que me preguntaron si tenía prácticas de riesgo, les decía que no, pero hasta ahí se quedaban, nunca era así ¿cómo sabes qué es una práctica de riesgo?** Segundo_Grupo_Nominal_Sesion01_P4_Hombre	*Tiempo de espera prolongado* **O4.** Laura: **Pues me dejaron esperando tenía el turno 12 que era la última yo, este esperé sin mentir, sin mentir tres horas** [ ]. Para mí fue demasiado aburrido [ ] EP2_01_Mujer_IUC *Retrasos en la atención* **O5.** Armando: Me dijo [en la recepción] que **no estaban disponibles** [los servicios amigables], que, porque la doctora no estaba, **y qué pues solamente, que volviera martes y jueves para ese tipo de cosas, especializada a jóvenes.** EP1_08_Hombre_ITS
Aceptabilidad/ adaptabilidad	*Atención en espacio inapropiado* **B1.** Laura: En su escritorio [ubicado en la sala de espera]] **me atendió y había pacientes que estaban esperando su consulta** […], y si me dio mucha pena hablar, **me dio mucha pena desarrollar mi papel** , […] me sentí muy cohibida **que las demás personas me estuvieran escuchando.** EP2_07_Mujer_IUC *Uso de material didáctico en la consulta* **B2.** Julio: Pues **era un consultorio un poco amplio, tenía varios carteles** y como artículos para que se hicieran las dinámicas, tenía sobre anticonceptivos, aparatos reproductores, o sea **si estaba como apto para dar una explicación de lo que sea** . EP4_09_Hombre_IVIH	*Resguardo de la confidencialidad* **B3.** Laura: “ **No creas que yo voy a ir corriendo y decirles a tus papás** que tienes relaciones sexuales ni nada por el estilo, **tú al venir aquí todo es confidencial y no sientas pena** ” […] EP2_11_Mujer_IUC
Seguridad	*Información completa y clara* **S1** . Claudia: **Solamente sacó el condón** , de hecho, no tenía femenino, pero me explicó con el mismo condón de hombre, y **fue con sus manos** o sea no tuvo como un maniquí [ ], **pero si me explicó** . EP3_09_Mujer_IMA *Fallas en la exploración física* **S2.** Armando: Y entonces **no fue una atención muy padre porque no me pesó, no me midió, nada** . EP1_01_Hombre_ITS *Negociación del uso de condón* **S3.** Armando: **Me dijo que hablara con la chica con la que estoy, […] y que le dijera** “tal vez yo sé que a ti te gusta que lo hagamos sin protección **tenemos que usar eso [condón]** más que nada para protegernos, para evitar un embarazo no deseado [ ]” EP1_06_Hombre_ITS	*Fallas en la información proporcionada* **S4.** Laura: […] **Ya me han enseñado muchas veces como se pone un condón y a ella como que se le fue uno que otro detallito al enseñarme** . Y si lo noté porque ya me lo quedé grabadísimo [ ]. EP2_07_Mujer_IUC **S5.** Claudia: *No me ayudó como en gran cosa, digo si* **me explicó los anticonceptivos un poco pero no a fondo como otros** *y yo creo que* **si no hubiera ido ya a otros centros** *de salud […]* **yo creo que no hubiera entendido nada de lo que me dijo ella** . EP3_07_Mujer_IMA
Continuidad	*Posibilidad de regresar en el momento que sea necesario* **C1.** Julio: Pues **me dijo que cuando quisiera podía regresar por eso es servicios amigables y no había ningún problema, que estaban las puertas abiertas** . EP4_03_Hombre_IVIH	*Medio de comunicación entre prestador y paciente* **C2.** Julio […] **me dejó sorprendido que me invitó que me podía dar cualquier consulta de seguimiento en los lugares donde ella** , [ la psicóloga] **me brindó la confianza que puedo ir con ella al lugar donde ella este, me dejó su teléfono** . EP4_08_Hombre_IVIH *Oferta de diversos servicios* **C3.** Laura: Pero **me dijeron que, si yo quería, podía agendar cita en la recepción para el dentista y para el nutriólogo** . EP2_06_Mujer_IUC

^a^EP1= Perfil 1, EP2= Perfil 2, EP3= Perfil 3, EP4= Perfil 4

^b^01 a 11= Código del establecimiento de salud

^c^Sexo del participante

^d^ITS= infecciones de transmisión sexual, IUC= Información sobre uso de condón, IMA= Información sobre métodos anticonceptivos y IVIH= Información sobre VIH.

### Accesibilidad

En general, se reportó fácil acceso debido a la ubicación geográfica y amplia disponibilidad de transporte público con acceso a las unidades. Las clínicas con señalización interna adecuada permitieron ubicar fácilmente el servicio. Eso se percibió como agradable ya que no tuvieron que preguntar al personal por el servicio, cuidándose la confidencialidad de la visita. En la mayoría de los establecimientos, se brindó atención gratuita sólo para el SSAA (Testimonios A1 y A2, Tabla 1 ).

Cuando las unidades no tenían señalización o ésta era inadecuada, debían pedir informes sobre el servicio. Pero las indicaciones no fueron claras en ocasiones, provocando demoras en la atención. Se presentaron, en una minoría de casos, barreras administrativas para recibir atención: consulta condicionada a afiliación a seguro médico y cobro por consulta o método anticonceptivo (Testimonios A3 y A4).

### Oportunidad

El tiempo de espera resultó menor a 30 minutos en la mayoría de las unidades. Pese a ello, se encontró una clínica donde esperaron aproximadamente cuatro horas para recibir atención. Una participante señaló que ella sólo esperó porque estaba desempeñando el rol de usuaria simulada, aclarando que, de otra forma se hubiera retirado (Testimonios O1, O2 y O4).

La mayoría de las unidades no contaban con un mecanismo para citas o reprogramación de consultas, dejando abierta la posibilidad de volver cuando requieran el servicio, pero sin garantizar que al regresar serán atendidos (Testimonio O5).

El personal buscó la forma de brindar atención o dar información que solucionara el motivo de consulta del adolescente. Se registraron prácticas inadecuadas de detección de conductas sexuales de riesgo. Los usuarios simulados detectaron que el interrogatorio resulta incompleto y percibieron que algunos prestadores no se sienten cómodos al hacer preguntas sobre el comportamiento sexual a los adolescentes (Testimonio O3).

Aunque fueron minoría, hubo clínicas donde no se brindó atención aludiendo a razones como: no disponibilidad del SSAA, saturación del servicio, falta de personal, horario inadecuado o, simplemente, se dijo que no se podía brindar atención sugiriendo que regresaran otro día (Testimonio O5).

El centro exclusivo para adolescentes tenía un horario de atención ampliado (matutino y vespertino). Además, contaban con mecanismos de programación y control de citas, a diferencia de los centros no exclusivos, donde sólo se ofertaba el servicio por la mañana, no existiendo control de citas.

### Aceptabilidad/adaptabilidad

Todos los servicios contaban con consultorios para dar atención a los adolescentes (Testimonio B2). La mayoría de los usuarios simulados fueron atendidos en un consultorio, pero no siempre eran exclusivos del SSAA, o proporcionaban un ambiente de privacidad (Testimonio B1).

La actitud del personal resultó central para el proceso de atención. Una buena actitud fue definida como: amable, respetuosa, confiable, sonriente, escuchaban sin interrumpir y mostraban interés. Esto generó confianza y seguridad en los adolescentes, quienes afirmaron que recomendarían el servicio. Una mala actitud se definió como: poco cordial, grosera, hicieron gestos, miradas incómodas o emitieron juicios sobre las prácticas sexuales de los usuarios, se sintieron regañados, incomprendidos y, por lo tanto, no volverían.

La política de confidencialidad fue dada a conocer a los adolescentes de manera excepcional (Testimonio B3). Pese a ello, los adolescentes expresaron la creencia de que, cuando el personal mostraba buena actitud, su información sería resguardada, o amenazada cuando el personal mostraba una mala actitud. Tanto la privacidad como la confidencialidad se vieron vulneradas por la atención en sala de espera, atención simultánea de pacientes en un mismo consultorio, interrupciones durante la consulta y dejar la puerta abierta durante la atención (Testimonio B1).

En el centro exclusivo se hizo regularmente mención de la política de confidencialidad, se explicó que la información sólo sería revelada en los casos donde se pusiese en riesgo su vida, además se les habló sobre sus derechos sexuales, mientras que en los servicios no exclusivos esto no ocurrió.

### Seguridad

El entrenamiento de los usuarios les permitió identificar errores u omisiones en la información proporcionada, la cual no siempre fue veraz, útil u oportuna (Testimonios S4 y S5). Hubo variabilidad de acuerdo con el tema consultado, siendo mejor explicado el uso de métodos anticonceptivos y del condón, mientras que el más pobre fue la consejería sobre ITS/VIH (Testimonio S1). El uso de material didáctico como maquetas, material audiovisual y maniquís se percibió positivo, ya que facilitó el aprendizaje.

La oferta de estudios diagnósticos dependió del caso de consulta, principalmente para el caso de sospecha de VIH, en donde se ofertó con mayor frecuencia la realización de pruebas rápidas. La información proporcionada sobre estudios diagnósticos fue poco entendible y resultó confusa. La exploración física fue una práctica poco habitual (Testimonio S2). En los casos donde se realizó, constó de toma de peso, talla y presión arterial. En raras ocasiones, se revisaron oídos y ojos. La exploración física fue más completa en el centro exclusivo para adolescentes.

Los prestadores salud, independientemente del motivo de consulta, hacían énfasis a los usuarios en el uso de condón con la pareja como único método que evita embarazos no deseados e ITS/VIH (Testimonio S3).

### Continuidad

No existió continuidad del motivo de consulta a través de consultas de seguimiento, aunque se dejó abierta la oportunidad de regresar cuando ellos necesitaran atención (Testimonio C1). Los establecimientos no ofertaron servicios adicionales a los de SSR, pocos invitaron a los usuarios a acudir a pláticas o talleres de SSR. En el centro exclusivo, se brindó seguimiento y oferta de servicios como nutrición y atención dental.

Se presentaron dos casos de excepción donde los prestadores, preocupados por la salud de los usuarios, brindaron un número telefónico personal o información sobre sus horarios laborales para dar seguimiento al caso. Ese elemento fue altamente apreciado por los adolescentes (Testimonio C2).

La dimensión de continuidad presentó una de las diferencias importantes entre las clínicas no exclusivas y exclusivas para adolescentes, en las segundas se observó la oferta de otros servicios en la unidad de salud de manera consistente (Testimonio C3). En la Tabla 2 se resumen los principales hallazgos, así como las diferencias entre servicios exclusivos y no exclusivos encontradas por los usuarios simulados.

**Tabla 2:** Resumen de hallazgos centrales y diferencias entre clínicas con servicios exclusivos y no exclusivos, México 2018.

Dimensión	Accesibilidad	Oportunidad	Aceptabilidad/ adaptabilidad	Seguridad	Continuidad
Hallazgos centrales	Fácil acceso geográfico Señalización deficiente Servicio gratuito, en algunos casos condicionado	Tiempos de espera aceptables Prácticas de detección ineficientes Falta o deficiencia de mecanismos control de citas	Variabilidad del lugar de atención Falta de resguardo de la privacidad No siempre se respeta la confidencialidad	Variabilidad de información según tema y personal Falta de exploración física Pobre oferta de estudios diagnósticos Recomendación de negociación de uso de condón	Falta de seguimiento Pobre oferta de servicios complementarios
Diferencias entre servicios exclusivos y no exclusivos	No hay	Amplio horario de atención (matutino y vespertino) en centro especializado, centros no exclusivos no cumplen con horarios ofertados. Agenda y control de citas en centro exclusivo	En servicios exclusivos se les habla de la política de confidencialidad y sobre derechos sexuales a diferencia de servicios no exclusivos	Exploración física más completa en el servicio exclusivo	Seguimiento al motivo de consulta y oferta de servicios complementarios en el centro exclusivo, a diferencia de los centros no exclusivos Se invita a volver acompañado de la pareja o amigos en el servicio exclusivo

## DISCUSIÓN

Existen áreas de mejora para lograr que la calidad de la atención en los SSAA sea óptima y efectiva, particularmente en los SSAA no exclusivos. De acuerdo con la experiencia de los usuarios simulados, los servicios son accesibles geográficamente. Sin embargo, nuestro estudio no presenta desagregaciones por tipo de localidad, por lo que difiere de otros estudios que señalan difícil acceso en áreas rurales, como es señalado por Regmi et al^25^. La mayoría de unidades no tienen letreros con horarios, días, costo de atención, ni señalización del servicio, provocando que los usuarios recibieran indicaciones del personal, concordante con otros estudios que reportan falta de señalización e indicaciones poco claras del personal^16 , 17^.

En México, el servicio amigable es gratuito. Sin embargo, se presentaron casos en que trámites como, cobro y requisitos de afiliación representaron una barrera. El cobro por el servicio es una barrera importante muy documentada para el acceso a los servicios de salud^16^. Si los adolescentes incurren en costos por el uso de los servicios, esto reduce las probabilidades de que vuelvan a utilizar el servicio o a solicitar métodos anticonceptivos. Adicionalmente, los adolescentes evitan utilizar servicios a través del seguro médico familiar, por temor a que sus padres se enteren ^5^ .

El tiempo de espera no constituyó una barrera para la mayoría de las clínicas. Sin embargo, hubo excepciones que motivaron que los adolescentes quisieran retirarse y no regresar a esa clínica. Schriver señala que es necesario optimizar la espera pues constituye una de las principales limitantes para acudir a los servicios^26^, barrera que puede ser aún más determinante para los varones, para quienes la necesidad de un servicio rápido y directo es importante^27^. Fueron pocos los casos donde se negó la atención, como lo encontrado por De Castro et al y Sykes quienes señalan que los profesionales negaron la atención a los adolescentes y se les invitó a volver otro día^17 , 28^. Las clínicas no cuentan con mecanismos claros para programar citas, constituyendo un problema central para la continuidad. Invitar a regresar a los adolescentes no garantiza que sean atendidos al regresar, desaprovechando adicionalmente la oportunidad de ofrecer información y satisfacer las necesidades de salud de los adolescentes.

La falta de privacidad es la principal barrera que enfrentaron los usuarios al acudir a los servicios. Las unidades tienen espacios destinados para brindar atención, pero la infraestructura no siempre es óptima para garantizar la privacidad^16 , 17 , 29^. Los usuarios prefieren unidades con espacios exclusivos para adolescentes, pero la mayoría de las clínicas no los tiene. Villalobos et al especifican que el 78.3% de las clínicas no tiene espacios exclusivos siendo este elemento registrado en la literatura como central^16 , 17 , 29^, especialmente para los varones, quienes temen que su masculinidad sea menoscabada al ser expuestos ante su comunidad, respecto a la atención de SSR ^5^ .

La privacidad y confidencialidad son claves para evitar que los usuarios sintieran pena o falta de motivación de expresar sus dudas^28^. Por ello, constituyen elementos cruciales en la satisfacción que perciben los adolescentes, ya que se genera un ambiente de desconfianza cuando no están presentes estas características.

La exploración física no es una práctica habitual, elemento que demerita la calidad de los SSAA los cuales deben brindar atención integral, y sistematizar la exploración clínica durante la consejería^9^.

Los usuarios señalan la necesidad de brindar información clara, veraz y específica, así como en un estudio previo^28^. La importancia dada por el personal al uso del condón como un método de prevención de ITS y embarazos no planeados y el uso de material didáctico fueron importantes para la consejería. Eso favoreció una consulta dinámica y facilitó la comprensión de los adolescentes como lo ha señalado De Castro^28^.

No existió seguimiento al motivo de consulta en la mayoría de las unidades, aun cuando se requería hacer pruebas diagnósticas de ITS/VIH, aunque se invitó a regresar al usuario cuando lo necesitara. Salvo el caso del centro exclusivo, el resto de los establecimientos no promueve servicios complementarios entre sus usuarios.

Un hallazgo fundamental para la calidad percibida por los adolescentes es la actitud del personal. La mayor parte tuvo una actitud respetuosa, pero se encontraron casos donde el personal emitió juicios, críticas o tuvo conductas poco profesionales. Las actitudes negativas del personal han sido señaladas como una de las principales barreras para la calidad del servicio^16 , 17 , 30 , 31^.

Si bien los servicios exclusivos fueron percibidos como exitosos, al brindar atención amigable, los requerimientos de infraestructura y recursos limitan su réplica de forma generalizada. Los servicios amigables anidados en clínicas de población sin seguridad social son el esquema que prevalece en el estado. Los hallazgos del presente estudio señalan las áreas de oportunidad para éstos y documenta buenas prácticas de atención, de acuerdo con los recursos disponibles en cada clínica.

En este estudio, la metodología de usuario simulado permitió obtener información objetiva, a partir de un cliente ficticio capacitado y estandarizado^20 , 33^. Las experiencias de los usuarios simulados durante la búsqueda y obtención de servicios de salud generaron datos evaluativos, resultado de un ejercicio de comparación y contraste que puede diferenciarse del pobre conocimiento sobre los estándares de calidad de los usuarios reales. Eso genera evidencia valiosa para la discusión de los estudios de calidad desde la perspectiva de los usuarios^34^.

Nuestros hallazgos y los análisis expuestos son observacionales y no buscan identificar causalidad entre los fenómenos estudiados. La metodología realizó una valoración crítica y facilitó la observación sin alterar el comportamiento del prestador al saberse evaluado^35^.

No se hizo diferenciación por áreas urbanas y rurales, debido a la ubicación geográfica de los establecimientos y no se abordaron todos los casos posibles de consulta. Aunque se contó con dos usuarios simulados varones y dos mujeres, no se buscó específicamente comprender las diferencias relacionadas al género o a la preferencia sexual de los usuarios, sino a las dimensiones de la calidad del servicio. Este estudio contribuyó al identificar hallazgos que pueden pasar desapercibidos para un usuario común, debido a la falta de conocimiento sobre el tema consultado.

## CONCLUSIÓN

Si bien, la mayoría de los establecimientos visitados por los usuarios simulados refieren tener un servicio amigable, aun distan de cumplir con las características de amigabilidad de acuerdo con las recomendaciones internacionales. Este estudio reporta hallazgos importantes de la calidad durante el proceso de atención. Los servicios no siempre brindan una atención integral a los adolescentes, olvidándose de la prevención y continuidad de la atención una vez atendido el motivo inmediato de consulta. Además, aunque fueron pocos casos, aún se condiciona la atención al pago de los servicios. Debido a la evidencia en la literatura sobre disparidades en las experiencias y consecuencias del embarazo adolescente, es necesario que los servicios incorporen un enfoque de género que permita el uso activo de los servicios de salud sexual y reproductiva por parte de los varones.

Se encontraron diferencias entre los centros exclusivos para adolescentes y los anidados en clínicas no exclusivas. La percepción de los usuarios simulados reveló que la atención en los centros exclusivos es de mayor calidad y más completa al ofertar diversos servicios, independientemente del motivo de consulta. Si bien no es posible para el sistema de salud contar con servicios amigables exclusivos en todos los casos, es necesario hacer adecuaciones que permitan que los servicios actuales adopten estrategias que hagan posible que los servicios no exclusivos brinden un servicio comparable con los del centro exclusivo. Este fue mejor valorado por los adolescentes señalando que regresarían en caso de requerir atención. Es un hecho que ha aumentado el número de clínicas que brindan a los adolescentes servicios de salud, pero es necesario hacer mejoras para lograr una atención de calidad.

Se requieren estrategias para mejorar las buenas prácticas y la calidad de la atención en salud para adolescentes. Entre ellas, el desarrollo de actividades en las comunidades, involucrando a las escuelas y padres de familia, para atraer y captar a las y los adolescentes antes de que inicien la actividad sexual. Además, la sensibilización y capacitación a administrativos y al personal de salud, en la atención a los adolescentes, de forma que los prejuicios y normas sociales no permeen la atención que se le ofrece a esta población.
